# Deadly Aspiration Pneumonia Secondary to Superior Mesenteric Artery Syndrome

**DOI:** 10.1155/2024/5055948

**Published:** 2024-02-07

**Authors:** Rhea Akel, Iskandar Daou, Dany Jamal, Elham Hobeika, Rany Aoun, Georges Nawfal

**Affiliations:** ^1^Department of Medical Imaging, Saint Joseph Hospital, Saint Joseph University, Beirut, Lebanon; ^2^Department of Intensive Care Unit, Saint Joseph Hospital, Beirut, Lebanon; ^3^Department of Gastroenterology, Saint Joseph Hospital, Beirut, Lebanon; ^4^Department of Digestive Surgery, Hotel Dieu de France Hospital, Saint Joseph University, Beirut, Lebanon

## Abstract

Superior mesenteric artery syndrome (SMAS) is a rare and unusual disease, suspected clinically and confirmed radiologically. It represents a duodenal obstruction secondary to the impingement of the third portion of the duodenum between the abdominal aorta (AA) and the superior mesenteric artery (SMA) due to decreased intraabdominal fat. High morbidity and mortality rates are linked to missed or late diagnosis that can lead to complications, such as gastric perforation and gastric hemorrhage. We present the case of a 33-year-old man who was not previously known to have a SMAS, who presented to the emergency department with signs of septic shock, complaining of fever and respiratory symptoms for several days. Investigations showed aspiration pneumonia secondary to an upper gastrointestinal obstruction with signs of SMAS on a computed tomography (CT) scanner. Acute and rapid deterioration led to cardiac arrest and death. Through this article, we highlight the importance of early and correct diagnosis of SMAS which can sometimes be challenging, since no number is strictly diagnostic and radiological images must be interpreted in light of the clinical history and physical examination.

## 1. Introduction

Superior mesenteric artery syndrome (SMAS) is a rare and unusual disease. It is considered to be a form of chronic duodenal ileus [1] and represents a duodenal obstruction secondary to the impingement of the third portion of the duodenum between the abdominal aorta (AA) and the superior mesenteric artery (SMA) due to decreased intraabdominal fat [2]. Clinical symptoms raise suspicion for this diagnosis which can be confirmed by radiological testing [3]; however, the diagnosis remains challenging and the approach for SMAS diagnosis remains controversial [1]. It is known that a delayed or missed diagnosis is linked to high morbidity and mortality rates secondary to complications, making it a crucial differential to be considered when there is a duodenal obstruction, especially in the setting of rapid weight loss [3].

## 2. Case Presentation

A 33-year-old man presented to the emergency department (ED), complaining of worsening fever, dyspnea, vomiting, and productive cough for 5 days, and an acute onset desaturation. The patient suffers from schizophrenia and has been treated for more than 20 years by using several neuroleptic drugs. From about 6 months ago, his psychiatric condition was not well controlled by medication, and he started weight loss due to low food intake.

His vital signs were as follows: temperature of 39.6°, heart rate of 147 beats per minute, blood pressure of 100/50 mmHg, and oxygen saturation of 80% on room air. His body mass index (BMI) was 14.5 kg/m^2^ (6 months ago, it was about 19.42 kg/m^2^). Physical examination on admission showed a conscious, noncooperant cachectic and dyspneic patient, with tachycardia but no murmurs. Pulmonary auscultation showed bilateral wheezing and bronchial congestion. Abdominal examination revealed epigastric tenderness, and no urinary symptoms were mentioned.

**Figure 1:**
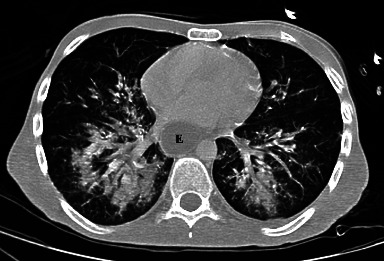
Axial CT scan of the chest with maximal intensity projection (MIP) reconstruction showing bilateral multifocal parenchymal condensations with multiple ground-glass opacities predominant in the lower lobes, suggestive of aspiration pneumonia. It also shows important distension of the esophagus (E).

Blood tests showed elevated white blood cell count (13,300/mm^3^) and a protein C level at 26 mg/l. The alkaline reserve was 12.1 mmol/l. Creatinine, liver function tests, sodium, potassium, and chloride levels were within normal limits. A computed tomography (CT) of the chest, abdomen, and pelvis with intravenous contrast material was ordered and revealed bilateral multifocal parenchymal condensations with multiple ground-glass opacities predominant in the lower lobes, suggestive of aspiration pneumonia (Figure 1).

**Figure 2:**
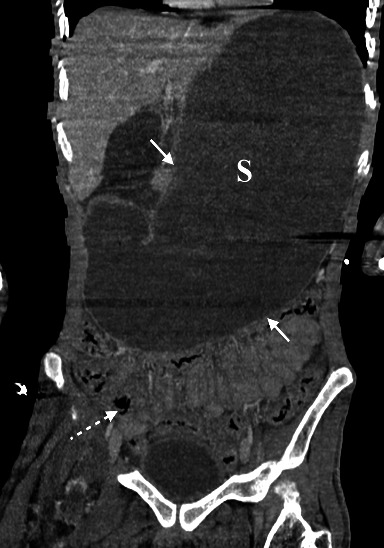
Multiplanar reconstruction (MPR) in the coronal plane shows important distension of the stomach (S white arrows), measuring 28 cm. The rest of the bowel loops shown are not distended (dashed white arrow).

**Figure 3:**
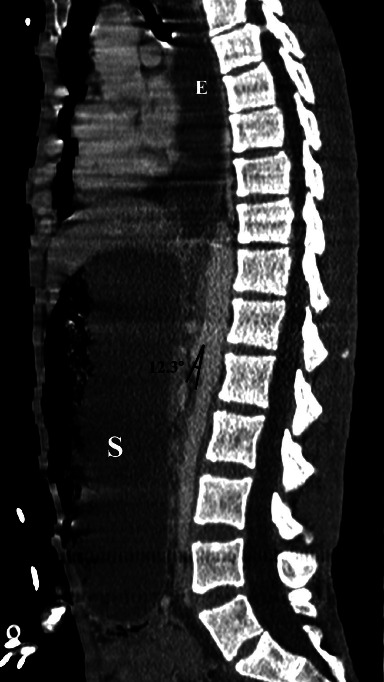
MPR in the sagittal plane shows an aortomesenteric angle of 12.3° and an extremely distended esophagus (E) and stomach (S).

**Figure 4:**
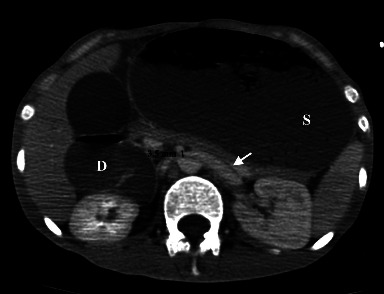
Axial CT scan showing an aortomesenteric distance of 3.5 mm, an extremely distended stomach (S) and duodenum (D), and a dilated left renal vein (white arrow) before the mesenteric clamp.

In addition, there was an extremely important distension of the esophagus, stomach, and proximal duodenum (Figure 2), that is, an upstream of an abrupt transition zone involving a decompressed caliber in the third part of the duodenum at the level of its crossing in the mesenteric clamp. The rest of the bowel loops were of normal appearance. No intraabdominal collection, parietal pneumatosis, or pneumoperitoneum were noted. The angle between the proximal SMA and the AA, also known as the aortomesenteric angle, was narrow, at 12.3° (Figure 3) and the aortomesenteric distance was 3.5 mm (Figure 4), suggestive of a SMAS. Also, the left renal vein was distended before its course in the mesenteric clamp.

Initial management included hydration, nasal oxygen, bronchodilators, nasogastric tube insertion, antipyretics, steroids, and hypercaloric total parenteral nutrition (2500 kilocalories per day). Empiric intravenous antibiotics (levofloxacin 500 mg daily and piperacillin/tazobactam 4.5 g QID) were started after blood and urine cultures. Evolution showed severe rapid clinical degradation that resulted in a cardiac arrest and death.

The diagnosis of a severe septic shock due to aspiration pneumonia secondary to SMAS was made.

## 3. Discussion

SMAS, also known as Wilkie's syndrome, was first described as a case report in 1842 by Carl Von Rokitansky and further detailed by Wilkie in 1927 [3, 4]. Other names for this syndrome include cast syndrome, chronic duodenal ileus, and aortomesenteric duodenal compression. It is a rare entity with an incidence estimated at 0.1%–0.3% [3]. This disease occurs preferentially in young adults and adolescents with an age range between 10 and 39 years, but it can occur at any age, and is more prevalent in females with a ratio of 3 females to 2 males [3]. No ethnic predisposition has been proven; however, familial cases have been reported [5].

The pathophysiology of this syndrome is explained by an important decrease of intraabdominal fat, causing a decrease of the intervening mesenteric fat pad between the AA and the SMA, resulting in a narrow aortomesenteric angle and distance [2]. The duodenal fat pad cushion is responsible for holding the SMA off the vertebral column and protecting it from duodenal compression [3].

In healthy individuals, the aortomesenteric angle ranges from 28° to 65° and the aortomesenteric distance ranges from 10 to 34 mm [6]. In SMAS, when the fat pad cushion becomes thin, the SMA leaves the AA at an abnormally hyperacute angle, typically less than 25°, and runs closer to the aorta, typically at a distance of less than 8 mm, depending on the BMI of the patient [2]. It is important to note that no number is strictly diagnostic, and radiological images must be interpreted in light of the clinical history and physical examination, which makes the diagnosis even more challenging [2].

Other characteristic findings on the CT scanner are distention of the stomach and proximal duodenum with bowel caliber narrowing at the origin of the SMA from the AA [7].

Our patient, in addition to being cachectic due to his psychiatric condition with a very low BMI of 14,5 kg/m^2^, had a hyperacute aortomesenteric angle and short aortomesenteric distance measuring 12.3° and 3.5 mm, respectively, with esophago-gastro-duodenal distension proximal with a transition zone at the takeoff of the SMA from the AA on the CT scanner. He was also treated with several neuroleptic drugs. Some of the known side effects of antipsychotic drugs are bowel hypoperistalsis and constipation [8].

Recent weight loss and symptoms of bowel obstruction should alert physicians to this condition. Late diagnosis has been linked to severe morbidity and mortality [3], emanating from electrolyte abnormalities, dehydration, malnutrition, gastric pneumatosis, portal venous gas, gastric perforation, and hemorrhage [9–11].

Our patient presented to the ED with signs of septic shock, pneumonia, and vomiting. The predominant distribution of the diffuse alveolar infiltrates, ground-glass opacities, and condensations in the lower lobes on the chest CT scan, has allowed us to make the diagnosis of aspiration pneumonia. In aspiration pneumonia, the right lower lobe is most frequently affected, and if aspiration occurs while in the prone position, both lower lobes might be involved [12].

Once the diagnosis is made, in the acute setting, conservative medical management is the initial treatment, including hydration, electrolyte correction, nasogastric tube insertion for gastric decompression, and total parenteral nutrition [3], which were all applied in our case. Nutritional support through hyperalimentation is added during conservative therapy in an attempt to increase the duodenal fat pad cushion, thus increasing the aortomesenteric angle and distance, thereby decreasing the duodenal compression and improving symptoms [3].

Albeit, many patients will fail the conservative measures and will require undergoing invasive procedures such as total parenteral nutrition, lysis of ligament of Treitz (strong procedure), transabdominal or laparoscopic duodenojejunostomy, percutaneous jejunostomy tube placement, and enteral tube placement [3, 7].

Our patient also received large broad-spectrum antibiotics based on a combination of levofloxacin and piperacillin/tazocin to treat the septic shock secondary to aspiration pneumonia.

The most common cause of death in SMAS is gastric perforation and hemorrhage [9–11]. Mortality due to aspiration pneumonia secondary to SMAS has not been described yet. Jung and Koo reported a case of a patient with SMAS, who presented with aspiration pneumonia secondary to induction of general anesthesia, and had a favorable outcome [13].

In our case, the patient received appropriate initial management for septic shock, duodenal obstruction, and SMAS, and the rapid clinical deterioration resulted in cardiac arrest and death.

## 4. Conclusion

SMAS is an unusual and challenging diagnosis that is made based on clinical suspicion and characteristic findings. Early and accurate diagnosis is a must to avoid serious complications that can lead to high morbidity and mortality rates. Gastric perforation and hemorrhage are considered the most common causes of death in SMAS. However, it is important to note that aspiration pneumonia in the setting of SMAS can be life-threatening when diagnosis is delayed.

## Data Availability

The data that support the findings of this study are available from the corresponding author upon reasonable request.

## Abbreviations

SMAS: Superior mesenteric artery syndrome
AA: Abdominal aorta
SMA: Superior mesenteric artery
ED: Emergency department
CT: Computed tomography
BMI: Body mass index.
